# An improvement project standardizing low prophylactic platelet transfusion dosing for infants

**DOI:** 10.1038/s41372-025-02347-5

**Published:** 2025-07-09

**Authors:** Kristen Coletti, Jennifer A. Hershey, Matthew Devine, Jennifer Taft, Jeff Schinella, Sekinah Ajiboye, Kathleen Gibbs, Michele P. Lambert, David Friedman, Christopher S. Thom

**Affiliations:** 1 Division of Neonatology, Children’s Hospital of Philadelphia, Philadelphia, PA USA; 2 Department of Pediatrics, University of Pennsylvania Perelman School of Medicine, Philadelphia, PA USA; 3 Center for Healthcare Quality and Analytics, Children’s Hospital of Philadelphia, Philadelphia, PA USA; 4 Data and Analytics, Children’s Hospital of Philadelphia, Philadelphia, PA USA; 5 Division of Hematology, Children’s Hospital of Philadelphia, Philadelphia, PA USA; 6 Division of Pathology and Laboratory Medicine, Children’s Hospital of Philadelphia, Philadelphia, PA USA

**Keywords:** Paediatrics, Adverse effects

## Abstract

**Background:**

Platelet transfusions are frequently given to preterm infants to prevent bleeding, but randomized trials demonstrated harmful effects from current practices.

**Problem:**

Many platelet transfusions were administered in 15–20 mL/kg doses.

**Methods:**

We sought to decrease platelet exposure among neonates by standardizing 10 mL/kg transfusions for non-bleeding thrombocytopenic infants in a level IV NICU.

**Interventions:**

We created evidence-based platelet dosing guidelines and changed practices in 3 plan-do-study-act cycles focused on education, reinforcement and electronic clinical decision support.

**Results:**

We reviewed 240 transfusions over 3 years. The percentage of 10 mL/kg transfusions improved from 17.6% to 100%, without increasing major bleeding and repeat transfusion rates. Monthly transfused platelet volumes decreased from 2269 ± 334 mL to 857 ± 181 mL (*p* < 0.001), conserving limited platelet resources and saving $2746–$4942 per month in platelets.

**Conclusions:**

This study improved our platelet transfusion practices and can facilitate similar transfusion guideline adoption to benefit neonates at other institutions.

## Introduction

Thrombocytopenia, defined as a platelet count <150,000/μL, affects 10–35% of infants admitted to the neonatal intensive care unit (NICU) [1–5], including approximately 70% of those with extremely low birth weight [6]. Platelets are frequently transfused prophylactically to thrombocytopenic infants with intent to prevent bleeding [6]. Unfortunately, bleeding often occurs independent of thrombocytopenia severity and regardless of whether platelets are transfused [7–12].

Mounting evidence demonstrates that platelet transfusions cause harm [1, 13–15]. The Platelets for Neonatal Transfusion Study 2 (PlaNeT-2) trial found that death or major bleeding were higher in infants randomized to receive platelet transfusion for counts <50,000/μL as compared to infants transfused using a more restrictive threshold of <25,000/μL [14], regardless of underlying disease severity [16]. Infants transfused at the higher threshold had more respiratory morbidity as well as higher neurodevelopmental impairment or death at 2 years of corrected age [14, 17]. Similarly, a retrospective analysis of an 819 infant cohort demonstrated higher rates of death and neurodevelopmental impairment among infants who received platelet transfusions [18]. Taken together, these data suggest that platelets are harmful when transfused to infants. Concerted efforts are needed to limit infants’ exposure.

There is wide practice variation regarding the appropriate platelet dose to transfuse. In the United States, platelet dosing guidelines do not exist. In European countries, guidelines recommend 5–20 mL/kg platelet doses [19]. Studies have shown that doses of 10 mL/kg and 15 mL/kg equivalently treat thrombocytopenia [20]. Importantly, these per-kg doses are high compared to transfusion practices in adults, where 1 unit of platelets corresponds to ~4 mL/kg [21]. Premature infants are sensitive to fluid shifts and have narrow or impaired autoregulation that increases intraventricular hemorrhage risk [22]. Attention to the potentially harmful effects of transfusion volume is needed [23].

Donated platelets are a limited resource, often in short supply and with and a restricted shelf life. Platelet storage is limited to 5–7 days, since most platelets are stored at room temperature to prevent premature activation. This increases donor exposure for neonates, as units cannot be retained for repeat transfusions like packed red blood cells. Initiatives preserving platelets and reducing donor exposure are necessary to optimize neonatal transfusion medicine.

Quality improvement (QI) initiatives standardizing restrictive platelet transfusion guidelines have been effective and safe. Davenport et al. [24] created a restrictive platelet threshold guideline for infants <6 months old and showed decreased platelet transfusion exposure. At our institution, a standardized reduction in prophylactic platelet transfusion dose for nonbleeding oncology patients was safely implemented without increased bleeding [25]. Stemming from this work, our multidisciplinary team designed a QI study to standardize 10 mL/kg prophylactic platelet transfusion dosing for nonbleeding infants in the NICU to reduce platelet exposure and unnecessary fluid administration. The specific, measurable, achievable, relevant, and timebound (SMART) aim was to increase the proportion of platelet orders with doses of 10 mL/kg from a baseline mean of 17.6% to >80% within 1 year. This article was written according to Standards for Quality Improvement Reporting Excellence (SQUIRE) 2.0 guidelines [26].

## Methods

### Context

This single-center QI initiative was conducted in a 100-bed Level IV NICU at the Children’s Hospital of Philadelphia in Philadelphia, PA. The NICU is part of a large academic medical center and serves as a regional referral center. Approximately 30% of the roughly 1200 infants admitted to the NICU each year are born prematurely at ≤34 weeks of gestational age. This is the specific population harmed by current platelet transfusion practices [14], although not all practices or transfusions are necessarily or overtly harmful.

All platelet ordering throughout the hospital is done within our electronic health record (EHR) (Epic^®^ Systems Corporation, Verona, WI) using customizable order sets. When ordering platelets, the ordering clinician must select an indication for platelet transfusion using a drop-down menu. All transfused platelets are apheresis-collected, pathogen-reduced platelet products stored in platelet additive solution (PAS) and plasma. All patients receive ABO-compatible cells.

Transfusions to infants were included in this initiative if the indication on the order was “thrombocytopenia without bleeding” in the EHR by the ordering clinician, irrespective of gestational age at birth. This means that we included transfusions given to preterm infants as well as full term infants. Infants were excluded if they had significant bleeding within the 72 h preceding transfusion, were on extracorporeal membrane oxygenation (ECMO), were on systemic anticoagulation, were having an invasive procedure (e.g., placement of an arterial line or peripherally inserted central catheter, lumbar puncture, cardiac catheterization, or surgery) within 12 h of the transfusion, or were <24 h post-surgery. Significant bleeding was defined as intraventricular hemorrhage, pulmonary hemorrhage, adrenal, liver, or gastrointestinal bleeding. These characteristics were ascertained using diagnosis codes in the EHR and confirmed by manual chart review.

### Interventions

**Fig. 1:**
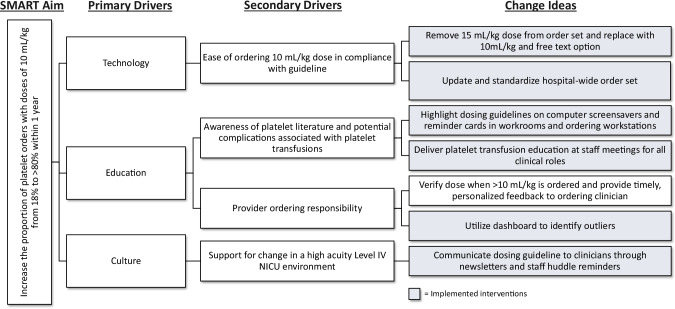
Driver diagram with the goal of increasing the rate of compliant 10 mL/kg platelet transfusion doses to >80% within a year of project inception. The primary and secondary drivers, and change ideas are described. Implemented change ideas are in gray colored boxes.

Our multidisciplinary team consisted of stakeholders from Hematology, Neonatology, and Transfusion Medicine. The primary team included 4 attending physicians, 1 neonatology fellow, 6 NICU front line clinicians, and clinical staff from the blood bank. The project had institutional support from a Quality Improvement Advisor and an informatics analyst who developed a data tracking tool. We initially developed a key driver diagram to plan interventions to achieve our aim of increased compliance with the 10 mL/kg platelet dose. Technology, awareness, and culture were identified as primary drivers (Fig. 1). We developed interventions corresponding to each of the drivers and implemented them sequentially in a series of 3 plan-do-study-act (PDSA) cycles.

Our initial PDSA cycle started in December 2022, focusing on unit-wide awareness of recommended dosing guidelines. A survey across our neonatal care network revealed that an evidence-based rationale for selecting a platelet dose was lacking at all practice levels [27]. Screensavers were posted with the recommended platelet transfusion dose and rationale on NICU computers for 90 days. This intervention targeted in-unit staff who document or place orders on these computers and were visible to all NICU clinicians. Additionally, we posted laminated memory aids with the recommended transfusion dose near all ordering clinicians’ workstations to specifically target this group. Simultaneous to this PDSA cycle, our Neonatology Division hosted a network-wide transfusion consensus conference recommending a threshold or trigger value platelet count of <25,000/μL for prophylactic transfusions [27].

We surveyed the ordering clinician representatives on the project team for feedback following the first PDSA cycle. Respondents reported awareness of the guideline and comprehension of the evidence underlying the new platelet dose recommendation. To sustain improved awareness throughout the unit, a second PDSA cycle beginning in April 2023 focused on reinforcement of education to supervising clinicians. This occurred via emailed clinical newsletters, monthly morbidity and mortality conference updates, and clinical meetings with attending physicians and fellows.

With evidence of short-term improvement following education-based interventions, our team identified a need for permanent workflow change with improved ease of ordering to sustain change. A third PDSA cycle, beginning in September 2023, targeted our EHR platform to customize platelet transfusion order sets. At project initiation, 15 mL/kg and 10 mL/kg were both dose options in the hospital-wide order set. We instituted clinical decision support where a platelet dose of 10 mL/kg automatically populated the dose field if a patient weighed <20 kg, although the defaulted dose could be changed at the discretion of the prescriber. Additionally, a rationale for ordering a 10 mL/kg dose to be run over 2 h for prophylactic transfusions in non-bleeding patients was listed in the order instructions. These order set changes were made hospital-wide after discussion with leadership from Neonatology, General Pediatrics, Pediatric Intensive Care, Cardiac Intensive Care, Oncology, Hematology, Emergency Medicine, Anesthesiology, and Surgical divisions, as well as approval from the Clinical Informatics Council, which is composed of informaticists representing care areas throughout the institution.

### Study of the interventions

Infants with platelet transfusion orders were identified using a graphic data display tool that captures ordering data from our EHR and allows for platelet ordering practice to be tracked in real time. This tool extracted platelet transfusion dose and patient weight, then calculated the primary metric of guideline compliance. We utilized statistical process control charts for project outcomes and balancing measures (Supplemental Fig. 1). The tool also extracted patient demographics and data relevant to each infant’s clinical course. Through the applications of filters for parameters such as indication for transfusion, anticoagulation, and procedures, it was possible to distinguish transfusions that met study inclusion and exclusion criteria.

We compared platelet ordering practices during the pre-intervention period using data collected retrospectively from the EHR from December 2021 through November 2022, with that from the intervention and subsequent sustain periods, where we prospectively collected data from the time of project implementation beginning December 2022 through August 2024.

### Measures

The primary outcome measure was compliant platelet transfusion orders with a dose of 10 mL/kg. Balancing measures were (1) repeat platelet transfusions within 36 h, to ensure that decreasing the dose did not result in more frequent prophylactic platelet transfusions, and (2) episodes of major bleeding within 72 h post-transfusion. Major bleeding was defined as any grade intraventricular/intracerebral hemorrhage, upper gastrointestinal bleeding, rectal bleeding, adrenal hemorrhage, or other non-specified hemorrhage. Episodes of major bleeding were identified using diagnosis codes in the EHR and confirmed via manual chart review. Our secondary outcome measure was the overall number and volume of platelet transfusions per month. Our tertiary outcome measure was cost associated with platelet transfusions. Given our goal of assessing unit-wide cost-savings, all platelet transfusions in the NICU were included in cost analysis.

### Analysis

Descriptive statistics, *t* tests and chi-squared tests were used to summarize and compare baseline characteristics and transfusion counts and volumes. Statistical process control p-charts were used to analyze our primary outcome measure and the balancing measure of repeat platelet transfusions within 36 h. Rational subgrouping with groups of 5 were used due to variability in the number of prophylactic transfusions that occurred each month. A g-chart was used to assess the balancing measure of major bleeding within 72 h post-transfusion given the rarity of bleeding events. We used c-charts to analyze changes in the number of transfusions and volume of platelet transfusions, normalized to patient-days. Charts were developed with QI Macros^®^ (Version 2024.10). Special cause variation was defined as a single point outside the control limits (astronomical point), ≥8 consecutive values above or below the centerline (shift) or ≥6 consecutively increasing or decreasing values (trend) [28].

We analyzed platelet transfusion use and conducted a cost analysis to assess savings related to practice changes. We compared total numbers of transfusion and transfusion volumes on a monthly basis over 3 epochs (baseline, test of change, and sustain periods). Cost calculations were based on a cost of ~$700 per unit of platelets from the American Red Cross. At our center, donated platelet units are 200–360 mL in volume. The difference in mean platelet volume used per month before and after the QI initiative were divided by 200 and 360 mL and multiplied by $700 per unit to create a range of estimated cost savings per month. This cost analysis was based on the direct costs of platelet units. Administrative costs were not considered, nor could we ascertain hospital-wide ordering practices or related platelet waste that would factor into cost savings realization.

### Ethical considerations

This project was completed as a Quality Improvement Initiative and was deemed exempt from oversight by the Children’s Hospital of Philadelphia Institutional Review Board.

## Results

### Implementation of platelet transfusion dose reduction guidelines

We tracked 240 transfusions among 66 infants. Demographics for patients in the pre-intervention and post-intervention groups did not vary substantially (Table 1). There was no difference in the mean gestational age at birth or day of life at transfusion (30.5 [26.8–37.3] versus 35.0 [25.6–37.0] weeks, median [interquartile range], *p* = 0.2). Post-intervention transfusions were earlier but not significantly different (day of life 27.0 [6.0–79.0] pre-intervention versus 21.0 [8.5–48.0] post-intervention, median [interquartile range], *p* = 0.3).

**Table 1:** Demographic characteristics of patients in this study.

Characteristic	Overall^a^	Pre-Intervention^b^	Post-Intervention^c^	*p-*value
Gestational Age (weeks)	34.0 (26.0–37.0)	30.5 (26.8–37.3)	35.0 (25.6–37.0)	0.2
Day of Life at Transfusion	23.0 (7.0–53.0)	27.0 (6.0–79.0)	21.0 (8.5–48.0)	0.3
Race				
White	31 (47)	13 (43)	18 (50)	0.6
Black or African American	12 (18)	5 (17)	7 (19)	0.8
Multi-Racial	9 (14)	6 (20)	3 (8)	0.2
Other	7 (11)	3 (10)	4 (11)	0.4
Indian	2 (3)	1 (3)	1 (3)	0.9
Asian	1 (2)	1 (3)	0 (0)	0.3
Unknown	4 (6)	1 (3)	3 (8)	0.4

Values represent median (interquartile range) or n (%). Statistical comparisons were made between pre- and post-intervention groups using Mann-Whitney or Chi-square tests. *P* < 0.05 was considered significant.

^a^*n* = 66 patients, 240 transfusions.

^b^*n* = 30 patients, 87 transfusions.

^c^*n* = 36 patients, 153 transfusions.

**Fig. 2:**
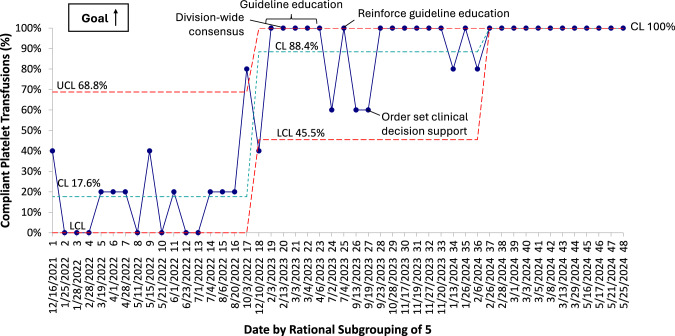
P chart demonstrating the percentage of compliant platelet transfusions with the recommended 10 mL/kg dose. Baseline data demonstrated 17.6% compliance. Special cause variation with center line shift was detected twice related to interventions (see annotations), increasing compliance to 88.4% during study period, and sustaining at 100% compliance. CL control limit, LCL lower control limit, UCL upper control limit.

During the pre-intervention period, 17.6% of platelet transfusion orders were of the 10 mL/kg dose (Fig. 2). Most doses were 15 mL/kg, reflecting common practice in line with the PlaNeT-2 trial dosing guidelines [14]. After initial education and awareness efforts (PDSA cycle #1), compliance with the 10 mL/kg dose increased (Fig. 2). Notably, this increase coincided with initial project discussions amongst key stakeholders, before the first PDSA cycle began. After PDSA cycle #2, a small decrease in 10 mL/kg dosing compliance was noted that did not meet criteria for special cause variation. A major improvement was noted after PDSA #3, which involved changing the EHR order set. We noted special cause variation with sustained process change that resulted in a centerline shift to an average of 88.4% after 7 months, surpassing our aim of 80% compliance (Fig. 2). A second centerline shift occurred, increasing dosing to 100% compliance after 6 months. This improvement was sustained in the subsequent 2 months, without further active intervention or group conversation about this topic.

### Platelet transfusion dose reduction did not increase bleeding or repeat transfusions

**Fig. 3:**
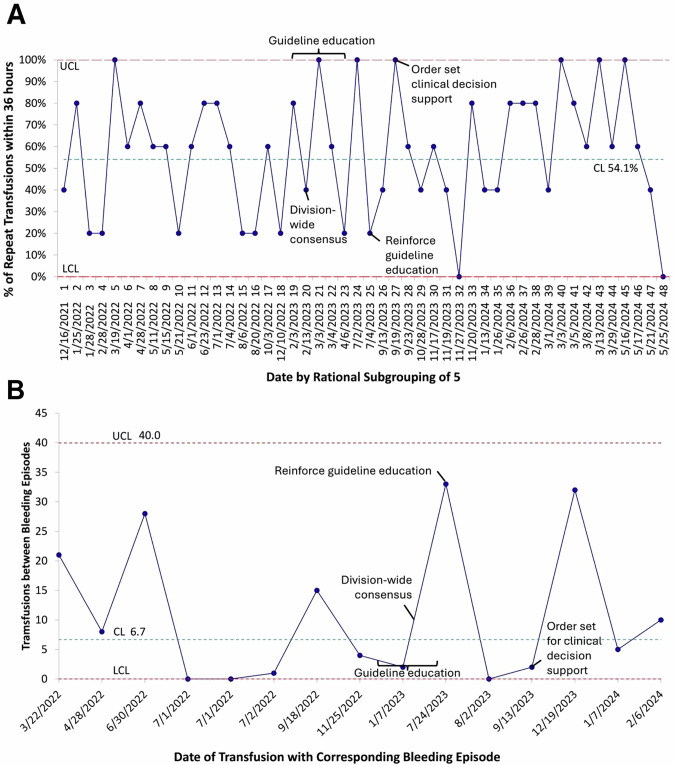
Balancing measures demonstrating that bleeding complications and repeat platelet transfusions were not affected by reduced platelet dosing. **A** P chart of repeat platelet transfusions within 36 h demonstrates no change in bleeding episodes with CL 54.1%. **B** G chart demonstrating 6.7 transfusions between bleeding episodes that remained unchanged. CL center line, LCL lower control limit, UCL upper control limit.

Both balancing measures were unchanged following interventions. Reduced platelet transfusion dosing did not increase bleeding complications. During both the pre-intervention and intervention periods, the mean proportion of infants with repeat platelet transfusions within 36 h was 54.1% (Fig. 3A). Transfusion frequency was not affected by lower platelet dose. The mean number of transfusions between bleeding episodes was consistent throughout the study (Fig. 3B).

### Cost savings attributed to platelet transfusion quality improvement efforts

**Fig. 4:**
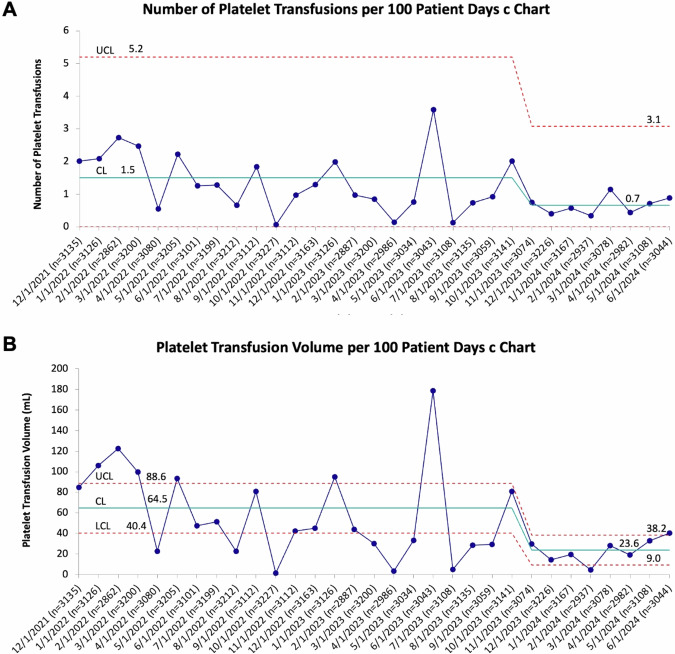
Platelet transfusion rates and volumes of platelets transfused in our NICU decreased over the study duration. **A** C chart depicting the number of platelet transfusions per 100 patient-days during the study period. Baseline data demonstrated 1.5 transfusions per 100 patient-days. Special cause variation with center line shift was detected once, decreasing the number of transfusions to 0.7 transfusions per 100 patient-days by study completion. **B** C chart depicting the volume of platelets transfused per 100 patient-days during the study period. Baseline data demonstrated 64.5 ml platelets transfused per 100 patient-days. Special cause variation with center line shift was detected once, decreasing platelet transfusion volume to 23.6 ml per 100 patient-days by study completion. The number of patient-days per month is shown along the x-axis (‘n’ values). CL control limit, LCL lower control limit, UCL upper control limit.

This initiative, along with platelet transfusion guideline establishment [27], changed platelet transfusion practices across our NICU. The number of platelet transfusions per 100 patient-days decreased by 50% over the course of our study across all NICU patients, prompting special cause variation (1.5 transfusions per 100 patient-days during study initiation vs 0.7 transfusions per 100 patient-days following sustained improvement, Fig. 4A). In the same time period, the volume of platelets transfused per month decreased by 63% and also prompted special cause variation (64.5 mL/month vs 23.6 mL/month per 100 patient-days, Fig. 4B). Patient census, as measured by patient-days per month, remained consistent over the study period (Fig. 4 x-axis ‘n’ patient-days per month). Measured on a monthly basis, a total of 2269 ± 334 mL platelets were given per month during the background period (December 2021–September 2022) vs 857 ± 181 mL/month during sustained improvement period (July 2023 – June 2024, mean ± SE, *p* < 0.001). In addition to limiting platelet exposure and volume for infants, these changes saved $2746-$4942 per month in platelets.

## Discussion

This quality improvement initiative demonstrates that a process of consensus guideline development, computerized clinical decision support, and education with reinforcement effectively changed platelet ordering culture and practice within a large level IV NICU. This initiative limited infants’ exposure to harmful platelets and unnecessary colloid administration [17, 18], without increasing bleeding complications or subsequent repeat transfusions. Fewer platelets were transfused after this initiative, resulting in unit-wide cost savings. We anticipate that these practice changes will ultimately reduce long-term morbidities in our patients [17], whether as a result of decreased volume loads and associated hemodynamic changes, or reduced delivery of adult platelets with inappropriate bioactivities [23].

The rapid and sustained improvements in dosing compliance met during this initiative reflect equipoise in the NICU community regarding platelet transfusion dosing. We found clinical teams readily amenable to practice change after education about recent evidence where greater platelet exposure was associated with harm [1, 13, 15, 17, 21].

Remarkably, 10 mL/kg platelet ordering compliance increased prior to our first formal PDSA cycle. Project discussion and meetings commenced three months prior, and about half of our multidisciplinary QI team were front line clinicians who place most platelet orders in our NICU. It appears that these discussions alone influenced platelet dosing practices (Fig. 2). Buy-in from this stakeholder group was imperative to the success of this initiative.

Our unit achieved 100% platelet transfusion dosing compliance ~5 months after PDSA cycle #3, which encompassed clinical decision support within the electronic medical record order set. This project took place in a high acuity, high volume, level IV center with hundreds of attendings, fellows, and front-line clinicians. Thus, widespread permeation was needed, and best sustained through concrete workflow support. The EHR order set change from a default 15 mL/kg to 10 mL/kg occurred across all hospital units, involving hospital-wide acceptance and collaboration. This default dose option does not preclude ordering clinicians from administering a higher dose. Resources from the Digital and Technology Services Department were needed to facilitate these changes.

We also noted direct institutional benefits from our transfusion practice changes. In conjunction with network-wide platelet transfusion guideline adoption [27], this initiative limited platelet transfusions in our NICU and saved an estimated >$2000 per month in donated platelet units alone ($2746-$4942, Fig. 4). This estimate likely underestimates total cost savings, as it excludes costs associated with administration of platelet transfusions that can dramatically exceed the costs of platelet products. The substantial direct cost savings may encourage adoption of similar guidelines in other institutions.

Bleeding risk was stable before and after our QI interventions, suggesting that a lower platelet transfusion dose did not modify bleeding risk. This aligns with neonatal studies where transfusing platelets in the setting of thrombocytopenia did not prevent bleeding [29]. Pediatric randomized controlled trials have also shown that platelet dose does not predict bleeding [30, 31].

Additionally, transfusion frequency was not affected with a lower platelet dose. Throughout the study period, 6.7% of infants received a repeat platelet transfusion within 36 h regardless of transfusion dose. This likely reflects the short half-life of exogenous platelets [21]. Longer term follow-up will determine if our interventions reduce IVH or other morbidities, including for high-risk preterm infants.

Although patient demographics in our NICU did not dramatically change during this study, we did note some differences in the day of life at time of transfusion in the pre- and post-intervention periods (Table 1). This resulted from a contemporaneous change in platelet transfusion guidelines that reduced the platelet transfusion threshold to <25,000/μL for prophylactic transfusions [27], which reduced use of prophylactic transfusions in older infants. Transfusions in older patients had caused a positive skew in the pre-intervention ‘day of life at transfusion’ estimates.

### Limitations

This study was undertaken in a large level IV NICU. Patients in our NICU may be of older gestational age (with lower IVH risk) than some delivery centers. Regardless, adoption of a 10 mL/kg transfusion dose should be widely applicable given evidence supporting decreased platelet exposure. This may be a less controversial practice change than adoption of platelet transfusion thresholds. Additionally, our cost-saving calculations are estimates based on estimated platelet costs, as data acquisition from patients included in this initiative was not possible. Future work is needed to evaluate the impact of our new practice guidelines on blood bank ordering practices at the institutional level to fully realize these cost savings.

We hope that our results and experience may facilitate adoption of similar dosing practices in other hospital systems, and potentially spur creation of national platelet transfusion guidelines. Platelet dosing guidelines in Europe range from 5 to 20 mL/kg [19], but no similar guidelines exist in the United States. Our results, based on literature-based guidelines from clinical trials, provide a convincing rationale to standardize prophylactic platelet transfusion dosing for non-bleeding NICU patients.

## Conclusion

Clinical trials have linked some platelet transfusion practices with harm in preterm infants, although platelet transfusions are undoubtedly necessary to prevent bleeding when demanded by clinical circumstances. While there are many potential etiologies for these findings, and variations in transfusion practices across institutions, limiting platelet exposure seems crucial to avoid transfusion-related morbidities and mortality. Our quality improvement study implemented a standardized 10 mL/kg platelet transfusion dose in critically ill infants within a level IV NICU using education, reinforcement, and clinical decision support strategies. Lower platelet dose facilitated preservation of a scarce resource and resulted in cost savings for our hospital, without influencing repeat transfusion frequency or bleeding episodes. Our experience may help other institutions adopt similar platelet transfusion guidelines.

## Article summary

We used quality improvement methods to implement 10 mL/kg platelet dosing for infants, saving costs and limiting platelet exposure without increasing bleeding complications or transfusion frequency.

## Supplementary information

SuppFig
