# Supplementary material for: An improvement project standardizing low prophylactic platelet transfusion dosing for infants
Source: J Perinatol. 2025 Jul 9;45(12):1825–32. doi: 10.1038/s41372-025-02347-5 (PMC12716995; doi:10.1038/s41372-025-02347-5)
Supplement: Supplementary file 1 — SuppFig [file 41372_2025_2347_MOESM1_ESM.pdf]

An Improvement Project Standardizing Low Prophylactic Platelet Transfusion Dosing for Infants

Kristen Coletti, MD[1,2], Jennifer A. Hershey, MPH[3], Matthew Devine BSc[4], Jennifer Taft, CRNP-NNP[1], Jeff Schinella PA-C[1], Sekinah Ajiboye CRNP NNP-BC[1], Kathleen Gibbs, MD[1,2], Michele P. Lambert, MD, MSTR[2,5], David Friedman, MD[2,6], Christopher S Thom, MD, PhD[1,2,*]

Affiliations:
[1] Division of Neonatology, Children's Hospital of Philadelphia, Philadelphia, PA, USA
[2] Department of Pediatrics, University of Pennsylvania Perelman School of Medicine, Philadelphia, PA, USA
[3] Center for Healthcare Quality and Analytics, Children's Hospital of Philadelphia, Philadelphia, PA, USA
[4] Data and Analytics, Children's Hospital of Philadelphia, Philadelphia, PA, USA
[5] Division of Hematology, Children's Hospital of Philadelphia, Philadelphia, PA, USA
[6] Division of Pathology and Laboratory Medicine, Children's Hospital of Philadelphia, Philadelphia, PA, USA

***Address correspondence to**:
Christopher S Thom
10-052 Colket Translational Research Building
3501 Civic Center Blvd
Philadelphia, PA 19104
267-760-7684
thomc@chop.edu

Supplemental Figure

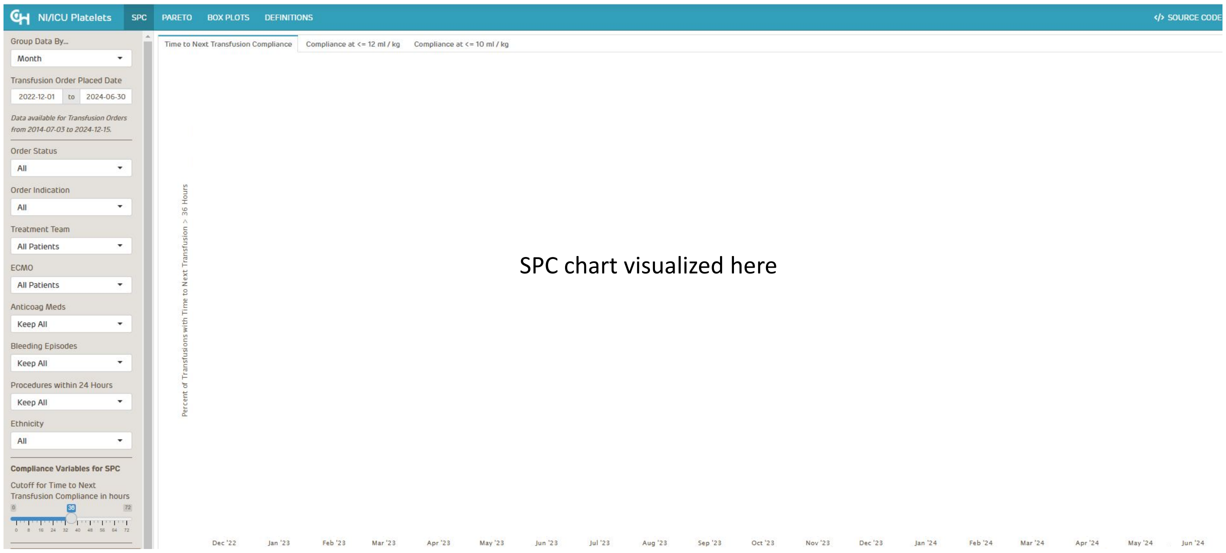

Supplemental Figure 1. Example of the clinical dashboard used to collect and monitor clinical transfusion practices during and after this initiative.
